# Causes of death in adults living with HIV in South Africa: A single-centre postmortem study

**DOI:** 10.4102/sajhivmed.v26i1.1673

**Published:** 2025-05-09

**Authors:** Tanvier Omar, Nadia Sabet, Alistair Calver, Gajendra Chita, Lucas E. Hermans, Willem D.F. Venter, Adriaan Basson, Monique Nijhuis, Annemarie Wensing, Neil Martinson, Maria Papathanasopoulos, Ebrahim Variava

**Affiliations:** 1 Department of Anatomical Pathology, Faculty of Health Sciences, University of the Witwatersrand, Johannesburg, South Africa; 2 Perinatal HIV Research Unit (PHRU), Faculty of Health Sciences, University of the Witwatersrand, Johannesburg, South Africa; 3 Department of Internal Medicine, Faculty of Health Sciences, University of the Witwatersrand and Klerksdorp-Tshepong Hospital Complex, Klerksdorp, South Africa; 4 Department of Medicine, Faculty of Health Sciences, University of Cape Town, Cape Town, South Africa; 5 Department of Medical Microbiology, Translational Virology Research Group, University Medical Centre Utrecht, Utrecht, the Netherlands; 6 Wits Ezintsha, Faculty of Health Sciences, University of the Witwatersrand, Johannesburg, South Africa; 7 HIV Pathogenesis Research Unit, Faculty of Health Sciences, University of the Witwatersrand, Johannesburg, South Africa; 8 Johns Hopkins University Center for Tuberculosis Research, Johns Hopkins University, Baltimore, United States; 9 Infectious Diseases and Oncology Research Institute, Faculty of Health Sciences, University of the Witwatersrand, Johannesburg, South Africa

**Keywords:** causes of death, HIV, suppressed, unsuppressed, immune reconstitution, immune reconstitution

## Abstract

**Background:**

Mortality among people living with HIV (PLWH) in developing settings remains elevated, despite high coverage with antiretroviral therapy (ART), with 70% – 80% being virally suppressed (VS).

**Objectives:**

This study aimed to determine cause-specific mortality in PLWH in South Africa.

**Method:**

An autopsy study with detailed medical record review was undertaken in PLWH dying in hospital. Minimally invasive autopsies were performed on 38 VS and 21 unsuppressed PLWH (≥ 18 years) dying in hospital between May 2018 and April 2022. We assessed clinical and histological findings to determine underlying, contributing, and immediate causes of death (CODs).

**Results:**

Median CD4 counts were 180 and 42 cells/mm^3^ in patients with and without VS respectively. Leading immediate CODs in both VS and unsuppressed PLWH were respiratory failure, sepsis, and septic shock; leading contributing CODs in decreasing order of frequency in both groups were acute kidney injury (AKI), bacterial pneumonia, immunological failure, gastroenteritis and current tuberculosis. Leading underlying CODs in both groups were hypertension, current tuberculosis, malignancies, and chronic obstructive pulmonary disease. VS was associated with lower risk of septic shock and AKI.

**Conclusion:**

VS on ART appeared to reduce risk of death from specific pathologies. However, infections, multi-organ failure, non-AIDS-defining malignancies, and metabolic diseases remain important CODs. Incomplete immune reconstitution appears to be a key contributor to premature death.

**What this study adds:** This post-mortem study combined clinical record review, histological evaluation and clinicopathology conferences to accurately determine cause specific mortality in people living with HIV in the ART era. VS on ART reduced death but imcomplete immune reconstitution appears to be a key contribute to premature death.

## Introduction

South Africa, with 7.6 million people living with HIV (PLWH), and 160 000 new infections per annum, has the largest number of PLWH globally, and the largest antiretroviral therapy (ART) programme; 5.6 million people were receiving ART in 2023, of whom 69% were virally suppressed (VS).^1^ This programme is credited with reducing HIV-related deaths from a peak of 305 491 in 2006, by 70% to 85 796 in 2022,^1^ with estimated gains in life expectancy of an additional 8.9 life years, higher in women than men.^2^ However, reductions in HIV-associated mortality do not yet meet WHO targets.^3^

In the pre- and early-ART era, mortality was mostly because of advanced HIV disease (AHD).^4,5^ With increasing access to ART, causes of death (CODs) in PLWH globally, particularly in the developed world, have pivoted from opportunistic infections and AIDS-defining cancers to non-communicable diseases and non-AIDS-defining cancers and infections.^6,7,8,9^ Globally, life expectancy has improved; longevity in well-controlled VS PLWH is only 1–4 years less than the general population.^10,11^ Despite this epidemiological shift, longevity for PLWH in developing settings is substantially lower than matched seronegative counterparts,^12,13^ because of poor virological control, treatment interruption, and low CD4 counts at ART initiation.^9,12,13^ There are few data from high HIV prevalence settings describing causes of severe morbidity and mortality in PLWH on ART.

A recent survey found 91% of South African PLWH were on ART and 94% of those were VS,^14^ although modelling data suggests this may be overstated.^15^ At our study site in North West province, > 50% of in-hospital deaths occurred in PLWH, only 28.3% of whom were VS and who experienced death 20 years earlier than in HIV-negative patients.^13^ Risks for premature mortality in PLWH are multifactorial, attributed to insufficient uptake of ART, initiation of ART at low CD4 counts, and poor treatment adherence.^9,12,13^

Although there are multiple descriptions of CODs in virally unsuppressed (VU) adults with AHD from high HIV prevalence settings, cause-specific mortality in VS PLWH in these settings is inadequately reported.^4,5,6,7,9^ A better understanding of CODs whilst VS could direct interventions to further reduce mortality. However, there are substantial discrepancies in assigning COD clinically, compared to autopsy.^16^ The objective of this study, therefore, was to combine clinical review of VS and VU hospitalised adult decedents’ medical records with histological examination of post-mortem minimally invasive tissue samples (MITS) to accurately determine and compare CODs.

## Research methods and design

We used a combination of standardised record review and MITS findings to determine underlying, contributing, and immediate causes of in-hospital deaths in both VS and VU PLWH in a district in North West province, South Africa, anticipating that this combination would markedly improve accuracy of COD determination. This study was nested within a larger autopsy study – *the FIND study: Core needle biopsies to detect hidden HIV reservoirs in hard-to reach tissue compartments of well-controlled and uncontrolled HIV-positive patients,* which included PLWH ≥ 18years who died in hospital.^17^

After obtaining informed consent from next-of-kin, a modified autopsy using MITS techniques was conducted. Core biopsies of organs and samples of blood, bone marrow, cerebrospinal fluid and bronchoalveolar lavage fluid (BAL) were collected. During histological analysis of the first 12 MITS samples to confirm representativeness of sampled organ, pathology was detected, prompting this cause-specific mortality sub-study. The protocol was amended to add multi-organ core biopsies for histological evaluation after the first 12 participants were recruited. A standardised histological examination of samples was performed, and an expert clinical review of the decedent’s medical record undertaken, blinded to autopsy findings. Results were discussed at 11 clinicopathological conferences (CPCs) to reach consensus for CODs, the results of which are presented here.

### Recruitment procedures

The FIND study recruited adult PLWH who died during hospitalisation at Tshepong Hospital, in North West province, South Africa. Tshepong is a tertiary referral hospital serving a population of 803 301, with an estimated HIV prevalence of 19.4% in 15–49-year-olds in 2022,^18^ of whom 60.5% were on ART in 2021.^15,19^ Decedents were eligible, provided autopsy procedures could be initiated within 16 h of death to ensure viable DNA collection for the parent study. Participants were identified by screening deaths occurring in internal medicine wards. Post-mortem, written informed consent was obtained from next-of-kin in the presence of an HIV counsellor. HIV status was disclosed to the spouse or sexual partner of the deceased if not previously known to them, followed by an offer of HIV testing. However, if the next-of-kin was not the spouse or sexual partner, and was unaware of the deceased’s HIV status, the HIV status was not disclosed, and the deceased was excluded. Exclusion criteria of the parent study included known haematological malignancies, intracranial infections and inadequate ART treatment records. Those not on ART at time of death were required to be ART naïve or to have interrupted ART for ≥ 6 months, and were excluded if they had received any antiretroviral agents in the 6 months prior to death.

### Autopsy procedures

A single study physician performed all autopsies in the hospital mortuary. Core biopsies were obtained using a BARD Magnum biopsy instrument and 16-gauge needles (BARD, Covington, Georgia, United States).^20^ We sampled bone marrow, brain and lungs bilaterally, and heart, liver, spleen and both kidneys under ultrasound guidance. Excision biopsies of sonar-identified or palpable lymph nodes, and skin and subcutaneous tissue sampling were performed. BAL fluid was obtained by inserting a feeding tube through a small tracheal incision and bronchial flushing with 50 mL sterile normal saline followed by withdrawal. If tuberculosis (TB) was suspected prior to death, this fluid was submitted for Mycobacterial Growth in Tube automated liquid TB culture (Becton Dickinson and Company, Sunnyvale, California, United States) and Xpert MTB/Rif Ultra (Cepheid, Sunnyvale, California, United States).

Histology samples were fixed in formalin, and routine haematoxylin and eosin-stained sections cut after paraffinisation. Slides were examined and reported on by a single pathologist. Additional stains were performed as indicated by the pathology noted.

### Cause of death determination

A standardised process of determining COD was devised prior to the first autopsy. A retrospective patient file review was undertaken independently by each of three senior specialist physicians in the Department of Internal Medicine; they reviewed in-patient records, including clinical notes, antemortem bloodwork, microbiology and histology reports, radiographic imaging, electrocardiogram and echocardiogram findings, and available outpatient records. Each reviewer provided a summary including immediate, contributing, and underlying CODs using WHO guidelines before receipt of post-mortem findings.^21^ This data was presented and discussed at 11 CPCs over the course of the study. A final consensus of CODs for each decedent was reached by the team, also identifying diagnoses missed antemortem, and missed opportunities for diagnostic or therapeutic interventions that may have contributed to death.

We acknowledge HIV infection was the underlying COD (UCOD) in most decedents. However, for the purpose of this study it is not included in the analysis; rather, in these decedents, as per WHO guidelines, existing diseases initiating events leading to death were classified as UCODs. The final disease or condition resulting in death was classified as the immediate COD (ICOD). Significant conditions contributing to, but not resulting in, the UCODs were classified as contributing CODs (CCODs).^21^

### Study definitions

Analyses were stratified by VS at time of death, defined as a viral load ≤ 400 RNA copies/mL – the limit of detection reported at the time of recruitment – from a whole blood sample collected within 1 month of death, or at post-mortem. VU decedents had a viral load > 400 RNA copies/mL within 1 month of, or at, death, and were either ART naïve, or had interrupted ART for a minimum of 6 months.^22^

Immunological failure was a CD4 cell count ≤ 100 cells/mm^3^ without concomitant or recent infection.^23^ Body habitus was reported based on clinical observation, as normal, obese, or wasted. Current TB was defined as TB diagnosed ≤ 6 months before, during final admission, or at autopsy, irrespective of receipt of TB treatment during hospitalisation; prior TB was defined as TB diagnosed ≥ 6 months before final admission, with no clinical or laboratory features of TB at final admission or autopsy. Definite TB was defined as TB diagnosed by culture, Xpert MTB/RIF Ultra, histology, or by identification of acid-fast bacilli on any clinical or autopsy specimen. Possible TB was defined as clinical or radiological features suggestive of TB in the absence of laboratory confirmation.^24^ Acute respiratory failure, sepsis and septic shock were defined according to accepted medical definitions.^25,26^

Anaemia was defined as haemoglobin (Hb) < 12.0 g/dL in women and < 13.0 g/dL in men, and severity classified according to National Cancer Institute Hb thresholds into mild, moderate, severe, or life threatening.^27^ Renal impairment was diagnosed using estimated glomerular filtration rate (eGFR) < 60 mL/min/1.73 m^2^. Oxygen saturation < 90% was interpreted as hypoxaemia.^28^ Parenchymal iron overload was determined histologically.

### Data analysis

Descriptive statistics for continuous variables are reported as means with standard deviations or medians with interquartile ranges (IQRs) based on data distribution. Proportions are reported with 95% confidence intervals (CIs). Statistical significance was set at a *P*-value of < 0.05. Fischer’s exact and Mann-Whitney *U* tests were used to tests for differences in baseline characteristics. Odds ratios and 95% CIs for risk of death in VS and VU decedents were calculated using univariate logistic regression.

### Ethical considerations

This study (reference no.: 161113) was approved by the University of the Witwatersrand Human Research Ethics Committee (Medical), and the Tshepong Hospital Research Committee. Post-mortem, written informed consent was obtained from next-of-kin in the presence of an HIV counsellor. Decedents were not recruited if this carried a risk of HIV status disclosure to a non-sexual partner next-of-kin.

## Results

**FIGURE 1:**
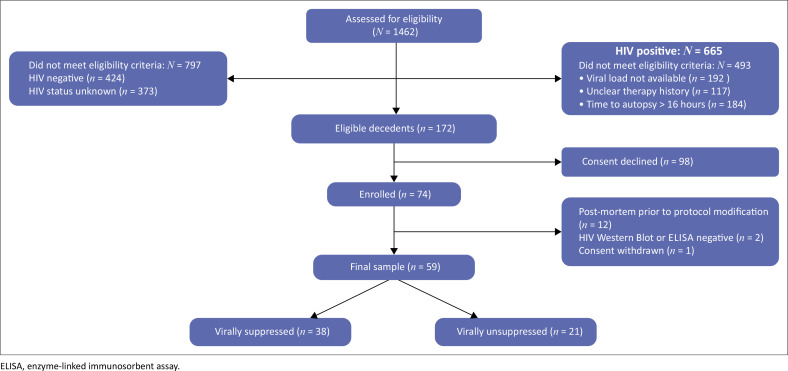
Flow diagram illustrating study enrolment process.

One thousand four hundred and sixty-two (1462) adult decedents in medical wards at Tshepong hospital between May 2018 and April 2022 were pre-screened; next-of-kin of 172 (11.8%) were approached for consent, of whom 74 (43.0%) consented (Figure 1). Exclusion was because of HIV serostatus unknown or negative, absent viral load results, inadequate ART history, or if > 16 h delay-to-autopsy was anticipated. The first 12 were recruited prior to commencement of this COD sub-study, two were excluded because of negative HIV Western Blots performed in two ART naïve decedents for uncertain HIV serostatus, and one consent was withdrawn prior to autopsy.

Of 59 decedents, 38 (64.4%) were VS (Figure 1); duration of suppression before death was available in 37, of whom nine (24.3%) were suppressed for < 1 month, nine (24.3%) for 1–5.9 months, one (2.7%) for 6–11.9 months and 18 (48.7%) for > 12 months. CD4 counts within 12 months of death were known for 33 VS decedents; 15 (45.5%) had CD4 counts of > 200 cells/mm^3^, nine (27.3%) had CD4 counts of 100 cells/mm^3^ – 200 cells/mm^3^, and nine (27.3%) had CD4 counts of < 100 cells/mm^3^. Similarly, CD4 counts within 12 months of death were available for 19 VU decedents and four (21.1%) had CD4 counts > 200 cells/mm^3^, four (21.1%) had CD4 counts 100 cells/mm^3^ – 200 cells/mm^3^, and 11 (57.9%) had CD4 counts of < 100 cells/mm^3^.

Male patients comprised 50% of VS and 66.7% of VU decedents (*P* = 022) (Table 1). The median age of VS decedents was older; they had a longer median duration since first HIV diagnosis and a higher nadir CD4 count than VU decedents. ART history was available for 35 VS decedents; most (74.3%) had been on ART for > 3 years, and 25.7% were on ART for > 1 year. Of the nine VU decedents on ART, three had been on ART for > 5 years, and one for > 1 year.

**TABLE 1:** Clinical characteristics of deceased participants undergoing minimally invasive tissue sampling, stratified by HIV viral suppression at death.

Clinical characteristic	Suppressed (*N* = 38)					Unsuppressed (*N* = 21)					Unadjusted OR†	95% CI
	*n*	Median	IQR	%	*n/N*	*n*	Median	IQR	%	*n/N*		
Age at death (years)	38	55.0	44–61	-	-	21	42.0	30–50	-	-	2.99	0.72–12.43
Male sex	38	-	-	50	19/38	21	-	-	66.7	14/21	0.50	0.17–1.51
HIV duration (months)	35	91.53	63.8–121.9	-	-	20	32.0	0.5–136.2	-	-	1.11	0.29–4.29
ART Duration (months)	35	67.83	10.0–98.1	-	-	4	76.0	36.2–102.4	-	-	2.15	0.40–11.44
Viral load at admission/ post-mortem (copies/mL)	36	0.0	0.0–0.0	-	-	18	121 096.0	2623–269 150	-	-	0.09	0.02–0.49
**Current / last ARV regimen**	38	-	-	-	-	21	-	-	-	-	-	-
Naïve	-	-	-	0	0/38	-	-	-	57.1	12/21	0.01	0.001–0.180
First-line	-	-	-	84.2	32/38	-	-	-	14.3	3/21	32.00	7.13-143.62
Second-line	-	-	-	15.8	6/38	-	-	-	14.3	3/21	1.13	0.25–5.05
Unknown	-	-	-	-	-	-	-	-	14.3	3/21	-	-
CD4 at death or <12 months (cells/mm3)	33	180.0	79.5–370.0	-	-	19	42.0	15–139	-	-	1.91	0.48–7.65
CD4 nadir (cells/ mm3)	36	160.5	48.0–333.5	-	-	19	42.0	15–139	-	-	1.53	0.39–5.99
Duration of suppression before death (months)	37	9.07	0.9–60.6	-	-	N/A	N/A	N/A	-	-	-	-
**Duration of symptoms** **prior to admission (days)**	35	5.0	2–14	-	-	17	7.0	2.5–24.5	-	-	0.46	0.12–1.82
Prior to tuberculosis	38	-	-	42.1	16/38	21	-	-	23.8	5/21	2.33	0.71–7.67
Respiratory symptoms present	38	-	-	44.7	17/38	21	-	-	61.9	13/21	0.50	0.17–1.48
Alcohol use	23	-	-	26.1	6/23	9	-	-	22.2	2/9	1.24	0.20–7.67
Smoking	23	-	-	56.5	13/23	9	-	-	55.6	5/9	1.04	0.22–4.91
Body habitus: Wasted	26	-	-	84.6	22/26	18	-	-	83.3	15/18	1.10	0.22–5.64
Body habitus: Obese	26	-	-	7.7	2/26	18	-	-	16.7	3/18	0.42	0.06–2.79
Hypertension	38	-	-	34.2	13/38	21	-	-	23.8	5 /21	1.66	0.50–5.57
Diabetes	38	-	-	10.5	4/38	21	-	-	0.0	0/21	5.61	0.29–109.44
Chronic lung disease	38	-	-	18.4	7/38	21	-	-	4.8	1/21	4.5	0.52–39.53
Abnormal chest X-ray	33	-	-	90.9	30/33	21	-	-	95.2	20/21	0.50	0.05–5.15
Decedents with anaemia	36	-	-	77.8	28/36	20	-	-	90	18/20	0.39	0.07–2.04
Oxygen saturation	30	92	84–98	-	-	18	92	84–98	-	-	1.23	0.31–4.82
Hypoxaemia	30	-	-	33.3	10/30	18	-	-	33.3	6/18	1.00	0.29–3.45
Decedents with eGFR < 60 mL/min per 1.73 m2 during admission	35	-	-	65.7	23/35	19	-	-	47.4	9/19	2.13	0.68–6.66
Median duration of admission prior to death (days)	38	6.4	1.3–11.5	-	-	-	6.4	1.3–11.5	-	-	1.11	0.29–4.29

CI, confidence interval; N/A, not applicable; OR, odds ratio.

† , odds ratios for medians of continuous variables calculated by binarizing values around group medians, performing a logistic regression using suppressed / unsuppressed classification as the target, and exponentiating found coefficients.

Thirty-two (84.2%) VS decedents were receiving the then South African guideline-recommended, first-line ART; the remaining six were on protease-inhibitor-based second-line therapy (Table 1). In VU decedents, 12 (57.1%), were ART naïve, six (28.6%) had defaulted treatment ≥ 6 months, and three (14.3%) self-reported being ART-compliant for ≥ 6 months at last admission. Date of first HIV diagnosis was available for 11 ART naïve decedents; eight were recently diagnosed a median of 0.39 (IQR: 0.15–0.74) months prior to death, and three at a median of 29.6 (IQR: 11.3–65.2) months. Treatment history was available for 6/9 unsuppressed decedents on ART: three had been on first-line and three on second-line therapy.

Overall, weakness, cough, shortness-of-breath, diarrhoea, vomiting, loss of weight and of appetite, in decreasing order of frequency, accounted for > 70% of presenting symptoms, with no difference between groups. Median duration of symptoms was 5.0 (IQR: 2–14) days in VS and 7 (2.5–24.5) days in VU. One quarter of the decedents’ medical records documented alcohol use (*P* = 0.74), and more than half were smokers (*P* = 0.95). The majority of participants in both groups were wasted. Over a third of VS and almost a quarter of VU decedents had hypertension (*P* = 0.41). Whilst anaemia was frequent in both groups, more VS decedents had mild or moderate anaemia (67.9% vs 55.6%; *P* = 0.40), whereas more VU had severe or life-threatening anaemia (32.1% vs 44.4%; *P* = 0.40). Almost all participants had abnormal chest x-rays, and one-third had hypoxaemia at admission. Renal dysfunction during admission was present in both groups (65.7% VS vs 47.4% VU; *P* = 0.20), but kidney failure was more prevalent in the VS (43.5% VS vs 55.6% VU; *P* = 0.40).

Brain, lung, liver and skin were adequately sampled, but kidney, bone marrow and fat samples were inadequate in 1.7% of MITS, and lymph node sampling in 4%; spleen was inadequate in 11.9% of the decedents, and heart in 14.3%.

### Clinicopathological conference consensus findings

**FIGURE 2:**
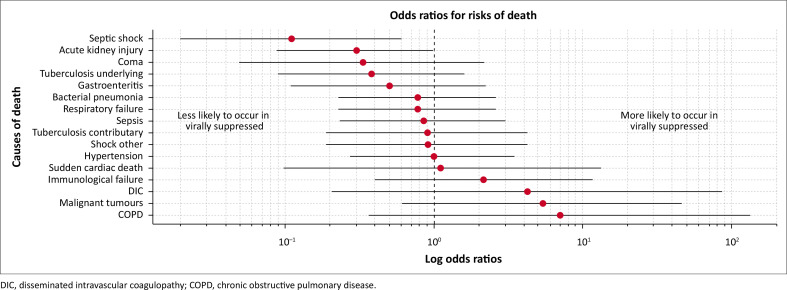
Log odds ratios for risk of death from leading immediate contributing and underlying causes of death in suppressed compared to unsuppressed decedents. The point estimates and 95% confidence intervals are shown for each cause of death.

Overall, the three leading ICODs, accounting for 62.7% of all ICODs, were: respiratory failure, sepsis, and septic shock (Table 2). However, the risk of septic shock in VS decedents was one tenth that of VU (Figure 2). Causes for sepsis or septic shock were pneumonia (13/22; 59.1%), urinary tract infections, including pyelonephritis (7/22; 31.8%), and gastroenteritis (2/22; 9.1%). In five decedents (22.7%), all VS, sepsis was a result of nosocomial infections, with mean duration of hospitalisation of 14.1 days (standard deviation: 12.8), longer than for those with community-acquired infections (7.8 days; standard deviation: 7.1) (*P* = 0.16).

**TABLE 2:** Immediate causes of death stratified by viral suppression, determined after the clinicopathological conference discussion.

Immediate causes of death (*N* = 71)†	All (*N* = 59)		Suppressed (*n* = 38)		Unsuppressed (*n* = 21)		OR	95% CI
	*n*	%	*n*	%	*n*	%		
Respiratory failure	15	25.4	9	23.7	6	28.6	0.78	0.23–2.59
Sepsis	13	22.0	8	21.1	5	23.8	0.85	0.24–3.04
Septic shock	9	15.3	2	5.3	7	33.3	0.11	0.02–0.60
Shock other‡	8	13.6	5	13.2	3	14.3	0.91	0.19–4.2
Coma	5	8.5	2	5.3	3	14.3	0.33	0.05–2.18
Disseminated intravascular coagulopathy	3	5.1	3	7.9	0	0.0	4.24	0.21–86.13
Sudden cardiac death	3	5.1	2	5.3	1	4.8	1.11	0.10–13.03
Other§	15	25.4	10	26.3	5	23.8	-	-

CI, confidence interval; OR, odds ratio.

† , Eight participants had 2 immediate causes of death, and 2 had 3 immediate causes of death;

‡ , Shock other: cardiogenic × 4, hypovolaemic × 2, obstructive × 1, Addisonian × 1;

§ , Other: Unknown 2; sudden unexpected death 2; 1 each: hepatic encephalopathy, intracranial event, status epilepticus, uremic encephalopathy, acute abdomen, acute kidney injury, immunological failure, severe anaemia, lactic acidosis, multi-organ failure, pneumonia.

We identified 115 CCODs in 59 decedents (Online Appendix 1: Table 1-A1), 62 in VS (mean: 1.6/decedent), and 53 in VU (mean: 2.5/decedent). Overall, the five lead CCODs, in decreasing order of frequency, were: AKI, bacterial pneumonia, immunological failure gastroenteritis and current tuberculosis (Table 3). AKI was the lead CCOD in VU (42.9%). For VS decedents one-third had the risk of dying with AKI compared to their VU counterparts (odds ratio [OR]: 0.30, 95%CI: 0.09, 0.99) (Figure 2).

**TABLE 3:** Leading contributing and underlying causes of death stratified by viral suppression (*N* = 59).

Causes of death	Total	%	Suppressed (*n* = 38)		Unsuppressed (*n* = 21)		OR	95% CI
			*n*	%	*n*	%		
**Contributing causes of death**								
Acute kidney injury	16	27.1	7	18.4	9	42.9	0.301	0.09–0.99
Bacterial pneumonia	15	25.4	9	23.7	6	28.6	0.780	0.23–2.59
Immunological failure	9	15.3	7	18.4	2	9.5	2.150	0.40–11.42
Gastroenteritis	8	13.6	4	10.5	4	19.0	0.500	0.11–2.25
Tuberculosis	7	11.9	5	13.2	2	9.5	1.440	0.2541–8.1539
**Underlying causes of death** †								
Hypertension	14	23.7	9	23.7	5	23.8	0.990	0.28–3.47
Tuberculosis	9	15.3	4	10.5	5	23.8	0.380	0.09–1.59
Malignant tumours	10	17.0	9	23.7	1	4.8	5.330	0.62–45.99
Chronic obstructive pulmonary disease	5	8.5	5	13.2	0	0.0	7.060	0.37–134.26

CI, confidence interval; OR, odds ratio.

† , Although all participants were PLWH, we did not classify HIV as the underlying cause of death.

We identified 117 UCODs in 59 decedents (Online Appendix 1: Table 2-A1), 76 in VS (mean: 2.0/decedent), and 41 in VU (mean: 2.0/decedent). Overall, the four lead UCODs were hypertension, current tuberculosis, malignant tumours, and chronic obstructive pulmonary disease (COPD).

Tuberculosis was diagnosed antemortem in 22/59 individuals (37.3%) but confirmed post-mortem as UCOD or CCOD in 17/59 (28.8%) decedents, representing 77.3% of those clinically diagnosed; no additional decedents were diagnosed with TB post-mortem. Of the VS decedents, 11/38 (28.9%) were diagnosed with TB antemortem and confirmed post-mortem in 9/11 (81.8%). The two without TB post-mortem had disseminated plasmablastic lymphoma, and bilateral bacterial pneumonia. Autopsy findings of the nine VS with confirmed TB, showed 4/9 (44.4%) had pulmonary, 3/9 (33.33%) miliary, and 2/9 (22.2%) isolated organ TB.

Of the VU decedents, 11/21 (52.4%) had a clinical diagnosis of TB antemortem, confirmed in 8/11 (72.7%) post-mortem. Three decedents thought to have TB antemortem were found at autopsy to have disseminated *Cryptococcus albidus* infection, bilateral actinomycotic pneumonia, and non-specific interstitial pneumonia with HIV-associated nephropathy.

The CPC identified pneumonia in 28/59 (47.5%) decedents; 16/38 (42.1%) were VS and 12/21 (57.2) VU (*P* = 0.27). On histological examination, 20/28 (71.4%) were bacterial, 6/28 (21.4%) viral, and 2/28 (7.1%) fungal in aetiology. VS decedents had significantly fewer opportunistic infections compared to their VU counterparts (55% vs 83%; *P* < 0.01).

Of 10 malignancies diagnosed, nine were in eight VS decedents (23.7%) (OR: 5.33; 95%CI: 0.62, 45.99). Men accounted for 4/9 (44.4%). Median age at death was 52 years (IQR: 43.5–65.5), and median ART duration was 39.9 months (IQR: 5.4–76.3). Four were lymphomas (two Non-Hodgkin and two Hodgkin lymphomas), and four were carcinomas (rectal and disseminated bronchogenic adenocarcinoma, oral squamous carcinoma and a disseminated poorly differentiated carcinoma of unknown primary). The remaining two were cutaneous Kaposi sarcoma, and a metastatic malignant tumour diagnosed clinically based on lytic skull and pelvic lesions.

Iron overload was a noteworthy underlying condition, identified exclusively in six VU decedents (28.6%). Being VS was associated with a 97% reduction in risk for iron overload (OR: 0.3; 95%CI: 0.002, 0.58).

Sepsis (12/17; 70.6%), bacterial pneumonia (11/20; 55%), AKI (10/17; 58.8%), and malignant tumours (5/10; 50%) were the most frequent conditions not diagnosed antemortem.

## Discussion

This is the first autopsy study we are aware of that compares CODs in VS decedents to those in VU PLWH. We made two important findings. First, septic shock and AKI were significantly less prevalent in VS individuals compared to VU. Second, despite most receiving ART, over half of VS PLWH fulfilled criteria for AHD. Other ICODs across the two groups were similar, leading with sepsis and septic shock, in line with published data reporting sepsis as the global leading cause of hospital admission and mortality, even in people on effective ART.^29,30^ However, VS decedents were over 10 times less likely to have septic shock than VU counterparts.

Over 50% of decedents in both groups smoked, increasing their risk for premature mortality.^31^ Despite effective ART, respiratory illness remains an important COD in VS PLWH. Bacterial pneumonia was a leading COD in both groups, as reported by others.^13^ As > 50% of VS decedents had a CD4 count < 200 cells/mm^3^, incomplete immune recovery appears to be an important risk factor. Although the proportion of decedents with definite TB was less than in prior studies,^4,5,6,7^ the proportions were similar in both groups, despite a 29-month median duration of suppression in the VS. Although ART with TB preventive treatment is efficacious in dramatically reducing both incident TB and death,^32^ we did not have data on receipt of preventive treatment. Surprisingly, TB in this study was diagnosed more frequently clinically than at autopsy. To our knowledge, this is the first autopsy study in which this is the case. High rates of TB and HIV co-infection in South Africa result in high indices of clinical suspicion, and initiation of empirical TB treatment. Our findings underscore the importance of laboratory confirmation of TB disease, especially in VS PLWH, and a low threshold for review where presumptive TB diagnoses are followed by poor treatment response.

AKI contributed significantly to death in both groups, but was a third less likely to in VS (OR: 0.30; 95%CI: 0.09, 0.99). Increased risk of AKI, with progression to chronic kidney injury and renal failure is frequently reported in PLWH.^33^ In VU PLWH, renal infections, nephrotoxic antimicrobials, hypovolaemia, sepsis, and tumours are common causes of AKI.^33^ As ART prolongs life, diabetes, hypertension, and pre-existing chronic kidney disease increasingly contribute to renal related deaths,^34^ and is the likely explanation for the high rates of hypertension (30.5%) and diabetes (10.5%) observed in our study, similar to others.^7,8,9,10^

The proportion of decedents with malignancies was high (17%), occurring predominantly in VS individuals, yet only 3/10 were AIDS-defining, consistent with reports of increasing non-AIDS related malignancies occurring as life expectancy increases.^8,9,10,35^

COPD was a common underlying cause of death in VS decedents. Others have reported COPD as an important respiratory cause of morbidity and mortality in PLWH, driven by smoking, increased pulmonary mucosal inflammation, and altered local immune responses to pathogens.^36^

Secondary parenchymal iron overload was found histologically in 28.6% of VU, but not in any VS decedents. Whilst both HIV infection and ART are associated with increased iron,^37^ our findings suggest a larger contribution is made by the former, whereas ART may be effective prevention. Others report an association of excess iron with enhanced HIV and TB replication, accelerated disease progression, and reduced survival.^37,38,39^

Anaemia present in both groups likely reflects the severity of HIV disease; a systematic review of anaemia in surviving PLWH reported a pooled prevalence of 46.6%,^40^ and an association with increased all-cause mortality, incident TB, and progression of HIV.^38^

Despite most being on ART for more than 3 years, many VS decedents did not have fully reconstituted immunity; fewer than half had CD4 counts > 200 cells/mm^3^, and 18.4% were clinically in immunological failure, likely contributing to premature death. Others have found increased mortality in VS PLWH at CD4 counts < 500 cells/mm^3^.^41^

We found missed opportunities for diagnostic or therapeutic intervention varied; some patients were not initiated on ART until several years after HIV diagnosis, and some with failing regimens were not switched timeously; there were multiple instances where actionable laboratory results were not responded to. Drug stockouts, insufficient community support for chronic illness, and prolonged wait times for investigations were also noted.

Our study limitations include biases inherent to all autopsy studies requiring informed consent; our single-site study may not represent PLWH dying in other high HIV prevalence settings and requires wider validation. Eligibility criteria for the parent study, particularly need for autopsy shortly after death and exclusion of CNS-related deaths, may have introduced selection bias and reduces generalisability. Detailed analysis of participants’ historical medical trajectory and its impact on COD was not undertaken. Our sample size is small, limiting power and statistical inferences, and odds ratios should be interpreted accordingly. Finally, MITS sampling may not always be fully representative, and pathology could be missed if not directly sampled.

## Conclusion

There is an association between being VS on ART and reduced death from opportunistic infections, septic shock, and AKI; however, we report increased non-AIDS defining malignancies and metabolic diseases in VS PLWH. In those on ART, incomplete immune reconstitution appears a key contributor to premature death. Improvements in prevention and diagnosis of TB, anaemia, and AHD are urgently needed. Moreover, if the longevity gap is to be reduced, test-and-treat strategies must be expanded for all PLWH, with the goal of starting ART when CD4 counts are high; ART programmes must ensure retention in care, with an emphasis on adherence and effective clinical monitoring.
